# miR-137 targets the inhibition of TCF4 to reverse the progression of osteoarthritis through the AMPK/NF-κB signaling pathway

**DOI:** 10.1042/BSR20200466

**Published:** 2020-06-15

**Authors:** Jinyu Wang, Liming Fang, Lili Ye, Shiliang Ma, Haoran Huang, Xiaoquan Lan, Jianlin Ma

**Affiliations:** 1 Department of Spinal and Joint Surgery, Qingdao Chengyang District People’s Hospital, Qingdao City 266109, Shandong Province, China; 2 Department of Radiology, Qingdao Chengyang District People’s Hospital, Qingdao City 266109, Shandong Province, China

**Keywords:** AMPK/NF-κB signaling pathway, miR-137, osteoarthritis, TCF4

## Abstract

**Purpose:** To explore the regulatory mechanism of miR-137 and transcription factor 4 (TCF4) in the progression of osteoarthritis (OA).

**Patients and Methods:** The expressions of miR-137 and TCF4 were detected in OA cartilage tissue, chondrocytes and OA rat cartilage tissue. miR-137 and TCF4 were up-regulated or down-regulated and transfected into chondrocytes and OA rat cartilage tissue. The gene expression, protein level, cell proliferation, apoptosis and inflammatory factors were detected, respectively. LPS and anterior cruciate ligament transection (ACLT) on the right knee were used to induce chondrocyte inflammation and establish rat OA model, respectively.

**Results:** miR-137 was low expressed in cartilage tissue of OA group, while TCF4 expression and protein level were significantly higher, showing significant negative correlation. In LPS group, chondrocyte activity was significantly inhibited, cell apoptosis ability was significantly enhanced, and the levels of inflammatory factors TNF-α, IL-1β, IL-6 were significantly increased. However, the above results were significantly improved after the up-regulation of miR-137 or down-regulation of TCF4. Double luciferase report revealed that miR-137 and TCF4 had targeted relationship. LPS induced activation of AMPK/NF-κB pathway and higher level of apoptosis. AMPK/NF-κB pathway inhibitor C could inhibit activation of this pathway, and up-regulation of miR-137 or down-regulation of TCF4 could significantly weaken the regulation of LPS on the pathway and apoptosis. Analysis of OA rat model showed that over-expression of miR-137 could inhibit up-regulation of inflammatory factors and activation of AMPK/NF-κB pathway.

**Conclusion:** miR-137 targets the inhibition of TCF4 to reverse the progression of OA through the AMPK/NF-κB signaling pathway.

## Introduction

Osteoarthritis (OA) is a chronic progressive joint disease, which will bring negative physical effects such as joint pain, tenderness and movement limitation to patients [1,2]. Epidemiological data of OA show that the disease affects approximately 14 million people in the United States, and the medical cost has increased by approximately 1.5 times. OA is more common in women, and it is one of the common causes of human disability in the world [3–5]. Its etiology is diverse. The joint injury, obesity, aging and heredity may increase its risk [6]. OA is a phenotypic heterogeneous disease. The key to the treatment of OA is to expand phenotypic applicability, suppress inflammation and degrade joint tissues [7,8]. Although the current treatment methods for OA include mesenchymal stem cell therapy, nanoparticle therapy and infiltration therapy, the efficacy lacks effectiveness and applicability, so the pathogenesis of OA still needs to be clarified [9–12]. Therefore, it is of great significance for us to explore new targets to curb the progression of OA by understanding the molecular mechanism of OA.

microRNA (miRNA) is a small RNA molecule that participates in basic biological processes. It can regulate genes and then regulate cell functions at the molecular level. At present, it has been found that it also participates in the regulatory network of OA chondrocytes. It has great diagnostic and therapeutic potential for OA [13–15]. In the researches of Soyocak [16] and others, miR-146a and miR-155 increased significantly in the progressive stage of OA, suggesting that they may be involved in the disease progression of OA. The researches by Li et al. [17] have indicated that miR-103 can increase the level of chondrocyte apoptosis and promote the disease process of OA by inhibiting SPHK1 activity and PI3K/AKT pathway activation. miR-137, the main character of our study, was previously reported to be involved in the disease process of osteoporotic fracture. Overexpression of miR-137 is helpful to reduce the fracture risk of osteoporosis patients. miR-137 is also a disease factor with differential changes in miRNA expression profiles of OA patients [18,19]. TCF4 has been reported to be targeted by miR-137 and involved in the molecular mechanism of colon cancer. In addition, TCF4 also mediates the lncRNA MFI2-AS1/miR-130a-3p axis regulatory network of OA induced by lipopolysaccharide (LPS), which plays a key role in the disease progression of OA [20,21]. AMPK/NF-κB pathway is a sensitive signaling mechanism involved in OA cartilage degradation and inflammatory response. Regulation of this pathway is beneficial to the prevention of OA [22].

At present, there is little research on miR-137/TCF4/AMPK/NF-κB axis regulatory network in the process of OA disease. We will conduct relevant exploration by detecting miR-137 and TCF4 expression and AMPK/NF-κB pathway related factors, hoping to provide reference for the containment and prevention of OA disease progress.

## Materials and methods

### Collection of tissue sample, cell culture and inflammation modeling

Patients were diagnosed with OA according to the American Rheumatology Association standard [23]. After total knee arthroplasty, their cartilages (*n*=34) were collected as OA group. In addition, tissues of traumatic amputees (*n*=26) were collected as normal group. The sample was collected from December 2016 to December 2018. All parties involved in the present study have signed informed consent forms.

Human chondrocytes (Zeye Biotechnology Co., Ltd., Shanghai, China, ZY-iCell-k004) were cultured at 37°C in 5% CO_2_ and DMEM medium containing 10% PBS (Yaji Biotechnology Co., Ltd., Shanghai, China, PM150312B). Inflammation modeling method [24]: LPS with a concentration of 100 ng/ml was used to induce the inflammatory state of cells for 1 h. In addition, compound C(C) [25] was pretreated for 1 h to verify the effect of AMPK/NF-κB signaling pathway in LPS-induced inflammation. The present study was reviewed and approved by Ethics Committee of Qingdao Chengyang District People’ Hospital and it was conducted according to the international guidelines of Helsinki Declaration.

### Cells transfection

The overexpression sequence (mimics) of miR-137, miR negative control (NC), targeted inhibition of TCF4 RNA(si-TCF4) and negative control RNA(si-NC) were respectively transfected into chondrocytes by Lipofectamine™ 2000 kit (Baimaige Biotechnology Co., Ltd., Wuxi, China, 11668019). The operation steps were strictly carried out according to the kit instructions.

### Establishment of OA rat model

Forty Sprague Dawley male rats (8 weeks old) (Fulbo Biotechnology Co., Ltd., Guangzhou, China) were reared in 12 h of light and 12 h of darkness, adapted to 7 days, and freely obtained food and water. The present study has been approved by the Animal Experimental Ethics Committee of Qingdao Chengyang District People’ Hospital. All methods were carried out according to the approved guidelines. The rats were kept in a specific incubator free of pathogens. Then, the rats were randomly divided into four groups: healthy control group (HC; *n*=10), sham operation group (sham; *n*=10), OA model group (OA; *n*=10) and OA model group intervened by minic (OA+minic; *n*=10). During the operation, all rats were anesthetized by inhalation of 2% isoflurane (Youcheng Biotechnology Co., LTD., Hong Kong, China, 709014). Anterior cruciate ligament transection (ACLT) was performed on the right knee of rats in OA group. The surgical incision was performed in the articular capsule of rats in Sham group, but not ACLT. The minic was injected into the articular cavity of rats after ACLT in OA+minic group. The sterile saline of equal volume was injected into rats in HC group. After operation for 20 days, rats in each group died from inhalation of 3.6% sevoflurane (Xiyuan Biotechnology Co., Ltd., Shanghai, China, XY-EP-Y0001046) in excess. The articular cartilage of medial tibial plateau was collected and stored at −80°C. The levels of miR-137, TCF4, p-AMPK, p-p65 and IκB-α were analyzed. Joint synovial fluid was collected, diluted with 2 ml sterile 0.9% NaCl, filtered with a 1.2 μm filter, mixed with 10% (v/v) protease and phospholipase inhibitor (Yuanye Biotechnology Co., Ltd., Shanghai, China, R40012), and centrifuged at 16,000×***g***  for 45 min at room temperature. The supernatant was collected and frozen at −80°C to analyze the levels of inflammatory factors. All animal work was performed at Qingdao Chengyang District People’ Hospital.

### qRT-PCR detection

Total RNA was extracted from articular cartilage samples and primary chondrocytes by TRIzol kit (Bioteke Biotechnology Co., Ltd., Beijing, China, RP2401). cDNA was synthesized by Bio-Rad Ssofast EvaGreen Supermixkit (Shanghai yihui Biological Technology Co., Ltd, China, 1725202) and real-time PCR system (Beijing Image Trading Co., Ltd., Beijing, China). All primers were designed and synthesized by Shanghai xinfan biotechnology co. LTD. miRNA used U6 as the internal parameters, and mRNA used β-Actin as the internal parameters. 2^−△△ct^ was used to analyze the data.

### Western blot analysis

RIPA lysate (Zhenyu Biotechnology Co., Ltd., Shanghai, China, PS0033) was added to cells in each group after culture to extract the total protein in the cells. BCA kit (Xinhua Lvyuan Technology Co., Ltd., Beijing, China, SS1175) was used to detect the protein concentration. The protein concentration was adjusted to 4 μg/μl, separated with 12%SDS-PAGE (Bangjing Industry Co., Ltd., Shanghai, China, BJ-RD965) electrophoresis and transferred to PVDF membrane. Then, 5% defatted milk powder (Lianshuo Baowei Biotechnology Co., Ltd., Shanghai, China, N/A433) was used to seal PVDF membrane (Xinhua Lvyuan Technology Co., Ltd., Beijing, China, IPVH00010) for 4 h. TCF4, p-AMPK, AMPK, p-p65, p65, IκB-α with dilution ratio of 1:600 and β-actin primary antibody with dilution ratio of 1:1000 were added and sealed overnight at 4°C. The first antibody was removed by washing the film. HRP-conjugated goat anti-rabbit second antibody (1:1000) was added and incubated at 37°C for 1 h. Antibodies were all purchased from Beijing fubo biotechnology co., ltd. ECL kit (Rongbai Biotech Co., Ltd., Shanghai, China, PW029-5 preps) and Bio-Rad ChemiDoc MP Imaging System (Yihui Biotech Co., Ltd., Shanghai, China, 17001402) were applied to detect chemiluminescence, and gray values were analyzed by Quantity One software.

### Detection of cells proliferation

MTT kit (Biolab Technology Co., Ltd., Beijing, China, SY0502-YQV) was used to evaluate the proliferation ability of chondrocytes. Cells were placed on a 96-well plate and grown at a cell density of 5 × 10^3^ cells per well. About 20 μl of MTT solution (5 μmg/ml) was added and cultured at 37°C for 4 h. About 200 μl of dimethyl sulfoxide was added to each well. GeneQuant 1300 spectrophotometer (Binzhi Biotechnology Co., Ltd., Shanghai, China) was used to measure the OD value of cells at 490 nm wavelength in each group.

### Detection of apoptosis ability

Transfected cells were digested with 0.25% trypsin (Fubo Biotechnology Co., Ltd., Beijing, China, TE2004Y) and washed twice with PBS after digestion. About 100 μl of binding buffer was added to prepare the suspension of 1 × 10^6^/ml. AnnexinV-FITC and PI were successively added and incubated at room temperature and in dark for 5 min. ACEA NovoCyte flow cytometer (Guangzhou Biocytocare Biotechnology Co.,Ltd., China, DLK0002051) was used for detection. The test was repeated for three times to take average value.

### Detection of inflammatory factors

The levels of inflammatory cytokines TNF-α, IL-1β and IL-6 in cell supernatant and synovial fluid were detected by enzyme-linked immunosorbent assay (ELISA) kits. The kits were purchased from Shanghai Guangrui Biotechnology Co., Ltd. The test was conducted in strict accordance with the kit instructions. Finally, the absorbance (OD value) at 450 nm in each well was read by M15 automatic enzyme labeling analyzer (Chenlian Biotechnology Development Co., Ltd., Shanghai, China) to calculate the levels of TNF-α, IL-1β and IL-6.

### Detection of double luciferase activity

Targetscan7.2 was applied to predict the target gene downstream of miR-137. TCF4 3′UTR-Wt, TCF4 3′UTR-Mut, mimics and NC were transferred into chondrocytes by Lipofectamine™ 2000 kit. The luciferase activity was detected by dual luciferase reporting kit (Chundu Biotechnology Co., Ltd., Wuhan, China, CDLG-4997).

### Statistical analysis

In this research, GraphPad 6 was used for data analysis and picture drawing. Independent sample *t*-test was used for comparison between the two groups. One-way ANOVA was used for comparison among groups, expressed as F. LSD-*t* test was used for pairwise comparison after the event. Multiple time points were expressed by repetitive measurement and analysis of variance, expressed as F. Bonferroni was used for post test. There was statistical difference with *P*<0.05.

## Results

### Expression of miR-137 and TCF4 in chondrocytes of OA patients

**Figure 1:**
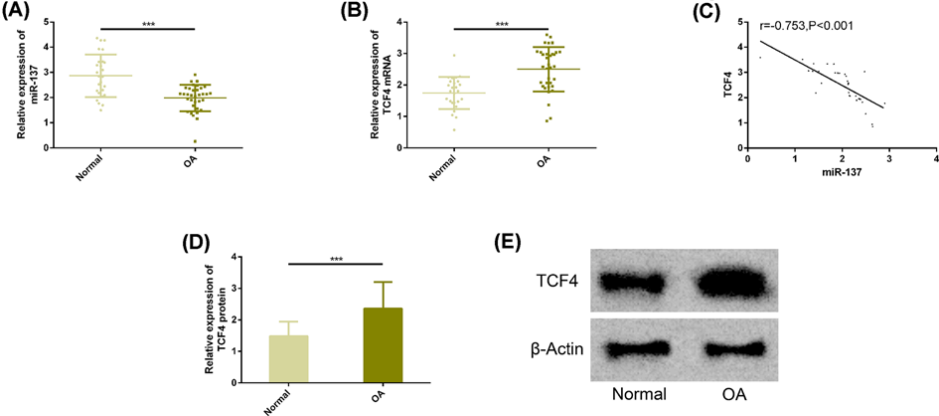
Expression of miR-137 and TCF4 in chondrocytes of OA patients (**A**) The expression of miR-137 was significantly lower in the tissues of OA patients. (**B**) The expression of TCF4 was significantly higher in the tissues of OA patients. (**C**) miR-137 had a significant positive correlation with TCF4. (**D**) The protein level of TCF4 was significantly higher in the tissues of OA patients. (**E**) Protein map of TCF4. Note: Compared between the two groups, * * * represents *P*<0.001. Abbreviations: miR, microRNA; OA, osteoarthritis; TCF4, transcription factor 4.

The results of real-time PCR analysis showed that the expression of miR-137 in tissues of OA patients was significantly lower than that of normal patients, while the expression of TCF4 was significantly higher. The differences were statistically significant (*P*<0.05). We further analyzed the correlation between miR-137 and TCF4, and found that there was a significant positive correlation between the two. Western blot analysis showed that the protein level of TCF4 in OA patients’ tissues was also significantly higher. More details are shown in Figure 1.

### Up-regulation of miR-137 can play a protective role against OA

**Figure 2:**
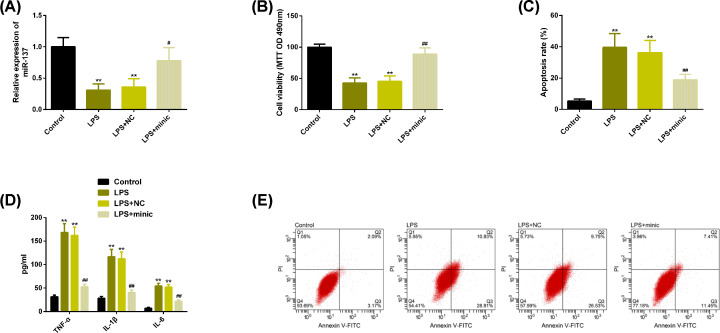
Up-regulation of miR-137 can play a protective role against OA (**A**) The expression of miR-137 in each group. (**B**) Up-regulation of miR-137 can reverse the significantly reduced cell proliferation ability under LPS intervention. (**C**) Up-regulation of miR-137 can reverse the significantly increased apoptosis rate under LPS intervention. (**D**) Up-regulation of miR-137 can reverse the significantly increased inflammatory factors under LPS intervention. (**E**) Flow cytometry. Note: Compared with the control group, **P<0.01; Compared with LPS group, ^#^*P*<0.05, ^##^*P*<0.01. Abbreviations: FITC, fluorescein isothiocyanate; IL-1β, interleukin-1β; IL-6, interleukin-6; LPS, lipopolysaccharide; miR, microRNA; NC, negative control; OA, osteoarthritis; PI, propidium iodide; TNF-α, tumor necrosis factor-α.

We used LPS to intervene chondrocyte to induce inflammation. Compared with the control group, the expression of miR-137 in LPS group and LPS+NC group was significantly lower, while the expression of miR-137 in LPS+minic group treated with high expression of miR-137 was significantly higher than that in LPS group, and the difference was statistically significant (*P*<0.05). We have observed the same results in chondrocyte proliferation, while we have observed significantly opposite results in cell apoptosis rate and the effect on inflammatory factors TNF-α, IL-1β, IL-6. More details are shown in Figure 2.

### Down-regulation of TCF4 can play a protective role against OA

**Figure 3:**
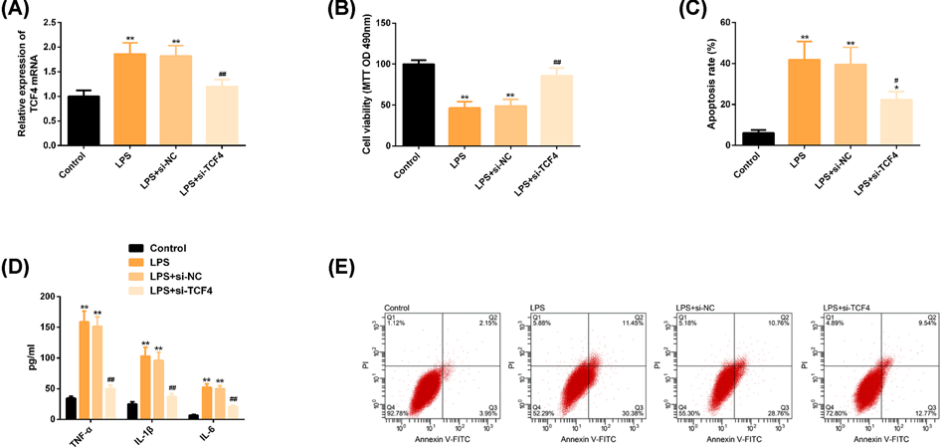
Down-regulation of TCF4 can play a protective role against OA (**A**) The expression of TCF4 in each group. (**B**) Down-regulation of TCF4 can reverse the significantly reduced cell proliferation ability under LPS intervention. (**C**) Down-regulation of TCF4 can reverse the significantly increased apoptosis rate under LPS intervention. (**D**) Down-regulation of TCF4 can reverse significantly elevated inflammatory factors under LPS intervention. (**E**) Flow cytometry. Note: Compared with the control group, **P<0.01; Compared with LPS group, ^#^*P*<0.05, ^##^P<0.01. Abbreviations: FITC, fluorescein isothiocyanate; IL-1β, interleukin-1β; IL-6, interleukin-6; LPS, lipopolysaccharide; NC, negative control; OA, osteoarthritis; PI, propidium iodide; si, short interfering; TCF4, transcription factor 4; TNF-α, tumor necrosis factor-α .

Compared with the control group, the expression of TCF4 in LPS group was significantly increased, chondrocyte activity was significantly inhibited, cell apoptosis ability was significantly enhanced, and the levels of inflammatory factors TNF-α, IL-1β, IL-6 were significantly increased. However, the above results were significantly reversed after down-regulation of TCF4, and the differences were statistically significant (*P*<0.05). More details are shown in Figure 3.

### Identification of miR-137 target genes

**Figure 4:**
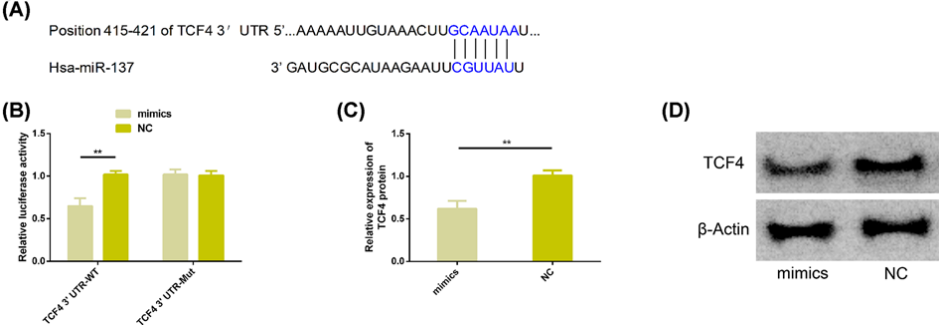
Detection of double luciferase activity (**A**) There was a targeted binding site between miR-137 and TCF4. (**B**) The relative luciferase activity-double luciferase report test. (**C**) The expression of TCF4 protein in transfected chondrocytes. (**D**) Protein map. Note: **P<0.01. Abbreviations: miR, microRNA; Mut, mutant; NC, negative control; TCF4, transcription factor 4; WT, wild-type.

We found that there was a targeted binding site between TCF4 and miR-137 through Targetscan7.2. The double luciferase activity detection results showed that TCF4 3′UTR-Wt luciferase activity decreased significantly after up-regulation of miR-137 (*P*<0.05), but it had no effect on TCF4 3′UTR-Mut luciferase activity (*P*>0.05). WB analysis showed that the expression of TCF4 protein was significantly decreased after transfection of miR-137-mimics (*P*<0.05). More details are shown in Figure 4.

### miR-137 protected chondrocytes by inhibiting AMPK/NF-κB pathway and apoptosis

**Figure 5:**
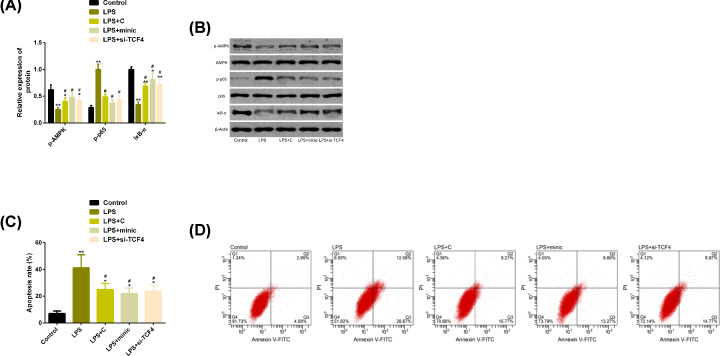
miR-137 protected chondrocytes by inhibiting AMPK/NF-κB pathway and apoptosis (**A**) Up-regulation of miR-137 or down-regulation of TCF4 can weaken the regulation of LPS on AMPK/NF-κB pathway. (**B**) AMPK/NF-κB pathway related protein map. (**C**) Up-regulation of miR-137 or down-regulation of TCF4 can inhibit the higher apoptosis rate of chondrocytes caused by LPS. (**D**) Flow cytometry. Note: Compared with the control group, *P<0.05, **P<0.01; Compared with LPS group, ^#^*P*<0.05. Abbreviations: AMPK, AMP-activated protein kinase; C, Compound C; FITC, fluorescein isothiocyanate; IκB-α, inhibitor of nuclear factor kappaB-α; LPS, lipopolysaccharide; miR, microRNA; NF-κB, nuclear factor-kappa B; p, phosphorylation; p65, transcription factor p65; si, short interfering; TCF4, transcription factor 4.

In the present study, AMPK/NF-κB pathway inhibitor C was used to verify whether AMPK/NF-κB pathway was involved in the protection mechanism of miR-137. Studies showed that LPS induced the activation of AMPK/NF-κ B pathway, which was manifested by phosphorylation of AMPK, significant reduction of IκB-α and significant enhancement of p65 phosphorylation. The differences were statistically significant (*P*<0.05). C inhibited the activation of this pathway. Similarly, up-regulation of miR-137 and down-regulation of TCF4 also weakened the regulation of LPS on AMPK/NF-κB pathway. In addition, C, minic, si-TCF4 also reversed the high apoptosis rate of chondrocytes induced by LPS. More details are shown in Figure 5.

### miR-137 inhibited the expression of inflammatory mediators and AMPK/NF-κB pathway in OA rats

**Figure 6:**
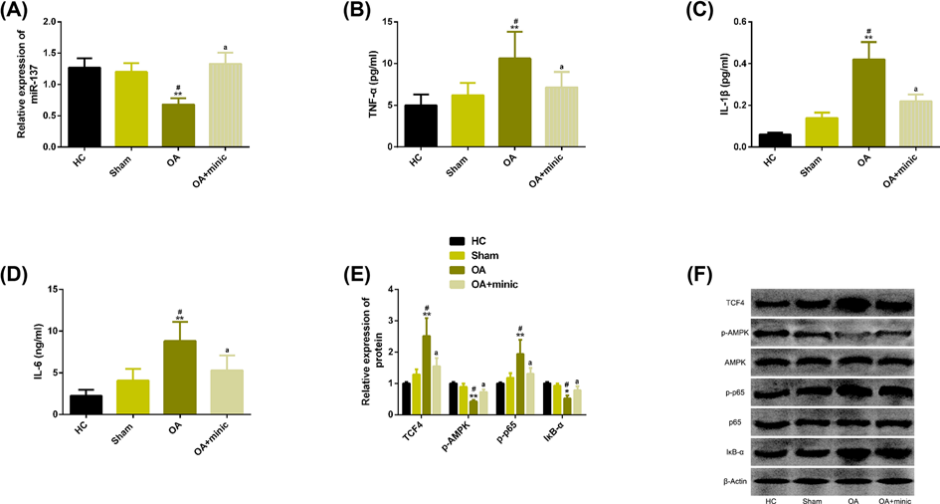
miR-137 inhibited the expression of inflammatory mediators and AMPK/NF-κB pathway in OA rats (**A**) Expression of miR-137 in articular cartilage of rats in each group. (**B–D**) Up-regulation of miR-137 can inhibit the levels of inflammatory factors TNF-α, IL-1β and IL-6 in OA rats. (**E**) Up-regulation of miR-137 can inhibit TCF4 and the activation of AMPK/NF-κB pathway in OA rats. (**F**) Related protein map of TCF4 and AMPK/NF-κB pathway. Note: Compared with HC group, **P<0.01; Compared with Sham group, ^#^*P*<0.05; Compared with OA group, ^a^*P*<0.05. Abbreviations: AMPK, AMP-activated protein kinase; HC, healthy control; IκB-α, inhibitor of nuclear factor kappaB-α; LPS, lipopolysaccharide; miR, microRNA; NF-κB, nuclear factor-kappa B; OA, osteoarthritis; p, phosphorylation; p65, transcription factor p65; TCF4, transcription factor 4.

We performed transection of anterior cruciate ligament of right knee in rats to establish OA model and verify the effect of miR-137 *in vivo*. The results showed that compared with the healthy control group, the expression of miR-137 in OA group was significantly reduced, and the levels of inflammatory factors TNF-α, IL-1β, IL-6 were significantly enhanced, while the above results were effectively alleviated after overexpression of miR-137. In addition, we also observed significant changes in protein levels of TCF4, p-AMPK, p-p65, IκB-α in OA group, and these changes could be inhibited by up-regulating miR-137. More details are shown in Figure 6.

## Discussion

More and more scholars have focused on the regulation of miR-137 and TCF4 on the process of OA, and published many research results. For example, in the research of Zhang et al. [26], miR-137 can regulate the growth, inflammation and extracellular matrix of chondrocytes through targeted inhibition of ADAMTS-5, thus inhibiting the progress of OA. It suggested that miR-137 was involved in the molecular regulation of OA disease process. Wu et al. [27] have pointed out that TCF4 is significantly positively correlated with the joint injury degree of OA patients, suggesting that TCF4 may be involved in the pathological process of OA. The reports by Xue et al. [28] have suggested that TCF4 is targeted at miR-93-5p, which mediates the activity and apoptosis of chondrocytes, and inhibition of its expression is helpful to alleviate OA progress. All the above studies have inspired us that miR-137 and TCF4 are probably involved in the regulation of OA disease process and play a key role in it. To this end, we conducted the following research to verify.

In the present study, we first detected the expression of miR-137 and TCF4 in cartilage tissue of OA patients. The results showed that miR-137 was significantly lower and TCF4 was significantly higher than that of the normal group, and the protein level of TCF4 was significantly higher, indicating that the two might participate in the pathological process of cartilage tissue lesions in OA patients. Next, our correlation results showed that miR-137 and TCF4 were significantly negatively correlated, suggesting that they might play antagonistic roles in the pathogenesis of OA. The pathogenesis of OA involves inflammatory state and apoptosis of chondrocytes, so we analyzed the above aspects at the molecular level [29]. We used LPS to induce inflammation of chondrocytes. In LPS group, rats showed lower level of miR-137 and higher level of TCF4, which was consistent with the previous tissue results. We found that under the intervention of LPS, chondrocytes showed the inhibition of cell activity, apoptosis level, and increased levels of inflammatory factors TNF-α, IL-1β, IL-6, while up-regulation of miR-137 or knockdown of TCF4 reversed the above results, suggesting that up-regulation of miR-137 or knock-down of TCF4 contributed to the improvement of the progression of OA.

We verified the relationship between miR-137 and TCF4 through double luciferase report. The results showed that the activity of TCF4 3′UTR-Wt luciferase decreased significantly after up-regulation of miR-137, but it had no effect on the activity of TCF4 3′UTR-Mut luciferase. Moreover, the protein level of TCF4 would be significantly decreased when miR-137 was up-regulated, indicating that miR-137 and TCF4 did have targeted regulation relationship. In AMPK/NF-κB signal transduction pathway, AMPK is a protein kinase related to the articular cartilage health and the regulation of OA process. The lack of AMPK will affect the articular stability of mouse chondrocytes and aggravate the development of OA [30]. NF-κB belongs to transcription factor family and it is closely related to inflammatory environment of chondrocytes and cartilage formation [31,32]. Therefore, we have also explored the regulatory mechanism of AMPK/NF-κB signal transduction pathway in OA. The results showed that LPS can induce activation of AMPK/NF-κ B pathway. Down-regulation of phosphorylated AMPK, IκB-α and up-regulation of p65 phosphorylation were typical characteristics. However, the activation signal of AMPK/NF-κ B pathway inhibitor C was significantly extinguished under the intervention of this pathway inhibitor C, and up-regulation of miR-137 or down-regulation of TCF4 also showed weakening of this pathway signal. It is understood that the phosphorylation levels of AMPK and p65 and the level of IκB-α protein are important indicators to measure the activation of AMPK/NF-κB pathway. The phosphorylation levels of AMPK and p65 are respectively related to the activation of AMPK and NF-κB. The activation of inflammatory signals of NF-κB needs to respond to the activation of AMPK pathway and the triggering of IκB-α [33,34]. In the present study, the activation performance of AMPK/NF-κB pathway marker is consistent with the previous research results [33–35]. In addition, the relatively high apoptosis level of chondrocytes induced by LPS was also significantly reduced under the above intervention, which suggested that miR-137 could prevent chondrocyte apoptosis by targeting down-regulating TCF4 and inhibiting the activation of AMPK/NF-κB pathway, thus inhibiting the progress of OA. At last, we also verified OA rats *in vivo*. Studies showed that up-regulation of miR-137 could inhibit the level of TCF4 protein, inflammatory factor and activation of AMPK/NF-κB pathway. This supported our results once again. Studies have reported that miR-137 can alleviate hepatic gluconeogenesis by regulating AMPK pathway, and it can also reduce β -amyloid induced neurotoxicity by inactivating NF-κB pathway [36,37]. In the studies of Fu et al. [38], the regulation of AMPK on process of regenerated myofibrosis is related to TCF4. In addition, Liu et al. [39] have reported that TCF4 can mediate the regulation of NF-κB pathway in lung cancer cells. Although both miR-137 and TCF4 are associated with the AMPK pathway or the NF-κB pathway, there are few reports about their regulatory effects on AMPK/NF-κB pathway.

All in all, we propose for the first time that miR-137 can reverse the disease process of osteoarthritis through targeted inhibition of TCF4-mediated AMPK/NF-κB signaling pathway, which may become a new direction for the treatment and prevention of OA. However, there is still room for improvement in this study. First, we can increase the prediction of miR-137 and TCF4 for different phenotypes and disability degree of OA patients. Second, we can increase the research on the potential anti-drug resistance mechanism of the two in the treatment of OA. In addition, we can supplement the research on the potential reaction of AMPK/NF-kB pathway to miR-137 and TCF4, and further expand the relevant regulatory mechanisms.

## Conclusion

All in all, we propose for the first time that miR-137 can reverse the disease process of osteoarthritis through targeted inhibition of TCF4-mediated AMPK/NF-κB signaling pathway, which may become a new direction for the treatment and prevention of OA. However, there is still room for improvement in the present study. First, we can increase the prediction of miR-137 and TCF4 for different phenotypes and disability degree of OA patients. Second, we can increase the research on the potential anti-drug resistance mechanism of the two in the treatment of OA. In addition, we can supplement the research on the potential reaction of AMPK/NF-kB pathway to miR-137 and TCF4, and further expand the relevant regulatory mechanisms.

## Abbreviations

HC: healthy control
IκB-α: inhibitor of nuclear factor kappaB-α
LPS: lipopolysaccharide
NF-κB: nuclear factor-kappa B
OA: osteoarthritis
TCF4: transcription factor 4
